# Do Variable Locking Plates Provide Better Functional and Radiological Outcomes in Volar Barton Fractures?

**DOI:** 10.7759/cureus.31427

**Published:** 2022-11-12

**Authors:** Karthik S J, Prabhu Ethiraj

**Affiliations:** 1 Orthopaedics, Sri Devaraj Urs Academy of Higher Education and Research, Kolar, IND; 2 Orthopaedics, Sri Devaraj Urs Medical College, Sri Devaraj Urs Academy of Higher Education and Research, Kolar, IND

**Keywords:** sarmiento's modification of lindstrom criteria, gartland and werley's demerit criteria, dash score, variable locking plate, volar barton fracture

## Abstract

Background: A volar or reverse Barton fracture refers to an intra-articular distal radius fracture that extends out to the volar aspect of the distal radius and there may be subluxation or dislocation in that direction. The overall goal of managing volar Barton fractures is to obtain motion that is pain-free, allow the patient to return to their usual activities, and minimize the risk of development of early-onset arthritis which may cause disability. Though various management options are available, this study deals with the assessment of functional and radiological outcomes of volar Barton fracture fixed with a variable locking plate.

Material and methods: A prospective, observational, and hospital-based study was conducted at R. L. Jalappa Hospital and Research Centre, Sri Devaraj Urs Medical College (SDUMC), Tamaka, India, on patients with volar Barton fracture managed with a variable locking plate from the period December 2019 to June 2021. Clinical data were assessed with Disabilities of Arm, Shoulder and Hand (DASH) score, Gartland and Werley demerit criteria, and Sarmiento modification of Lindstrom criteria.

Results: Forty subjects with a mean age of 36.43 ± 10.59 years were included in the study. The mean flexion, extension, supination, and pronation were 70.63° ± 3.6°, 74.3° ± 3.64°, 79.83° ± 2.67°, and 76.28° ± 1.99° degrees respectively at the end of six months. Based on Gartland and Werley's outcome, the majority (65%) of the study population was found to have an excellent outcome, 30% had a good outcome and only 5% had a fair outcome. The mean DASH score was 13.98 ± 5.76. According to Sarmiento's modification of Lindstrom criteria, 22 patients had good radiological outcomes, 11 had excellent outcomes and seven had fair outcomes.

Conclusion: The study concludes that fixation of volar Barton fractures with variable locking plates has a better role in immediate stability, maintaining anatomic reduction, and early mobilization. It also provides better functional outcomes and a good anatomical reduction.

## Introduction

Fractures of the distal radius are one of the most common fractures of the upper extremity seen in the emergency room. This type of injury has likely been common since humans existed but Petit, Pouteu, and Colles described it first as the injury might be due to fracture but not due to dislocation [1,2]. Barton’s fracture is titled after the American surgeon John Rhea Barton. The Barton fracture refers to an intra-articular distal radius fracture extending through the dorsal cortex of the radius, often with dislocation or dorsal subluxation of the radiocarpal joint. A volar or reverse Barton describes a fracture that extends out the volar aspect of the distal radius and there may be subluxation or dislocation in that direction [2]. Barton’s fractures account for 1.2% to 4.2% of all distal radius fractures. Based on the site and shifting direction of fragments they are classified as dorsal or volar Barton fractures [3].

According to AO classification, volar Barton fractures are classified under type B3 fractures of the distal radius. Conservative management options are frequently not effective and can result in complications such as osteoarthrosis, subluxation, deformity, and instability [3]. The treatment goal of volar Barton fracture is to achieve good reduction and stability which will help in early wrist mobilization and avoid complications [4]. Fracture healing depends on the following factors: blood supply, gap, and stability [5].

Various management options are documented in the literature, but internal fixation by open reduction using the volar plate system is currently used for fixation of volar Barton fracture as it helps in good reduction and provides better stability. Moreover, the patient can be mobilized early with less chance of developing joint stiffness [6]. Variable-angle locked plating has been developed to provide higher flexibility and the advantage of allowing fragment-specific capture or avoiding intra-articular placement of screws [7]. The compressive forces exerted on the bone are reduced by the locking plate usage and it helps in achieving stability and prevents the impairment of blood supply and periosteal compression which is favorable for the fracture union [8]. The locking screws support the underlying bone and the axial forces are resisted. The achievement of primary stability with a locking screw prevents the secondary displacement regardless of the bone quality. It also allows the patient early postop rehabilitation [9].

This study aims to assess functional outcomes of volar Barton fractures treated with variable locking plates by using the Disability of Arm, Shoulder, and Hand (DASH) score and Gartland and Werley demerit criteria, and radiological outcomes by using Sarmiento's modification of Lindstrom criteria. We also correlate the functional and radiological outcomes of volar Barton fractures fixed with a variable locking plate.

## Materials and methods

The Institutional Ethics Committee of Sri Devaraj Urs Medical College, Tamaka, gave approval in June 2019 with the approval number SDUMC/KLR/IEC/158/2019-20. Forty patients who presented to the emergency medicine department of R.L. Jalappa Hospital or the Outpatient Department of Orthopaedics with a history of self-fall, assault, or road traffic accidents (RTA) were considered for the study. Radiographs of the affected wrist were taken. The limb was immobilized with a below-elbow slab and fitness for surgery was taken. After informing about the risks and plan of management, written informed consent was taken. All patients received injectable antibiotics (cephalosporins) given one hour before surgery and continued postoperatively for five days. All the patients were advised to start making finger movements immediately in postop after the recovery of anesthesia. The sutures were removed two weeks after surgery. Rehabilitation was started to achieve gains in active and passive wrist movements. All the patients were followed up at one month, three months and six months.

Check x-rays were taken to assess fracture union and signs of failure of fixation. Functional outcome after surgery was assessed by using the DASH score and Gartland and Werley demerit criteria. Radiological outcomes after surgery were assessed by using Sarmiento modification and Lindstrom criteria. Patients aged between 18 and 60 years who presented with isolated closed volar Barton fracture were included in the study and patients with pathological fracture or with delayed presentation (>four weeks) were excluded.

Study design and sample size calculation

This was a prospective, interventional, hospital-based study. The sample size for the study was estimated on the basis of a number of cases likely to satisfy the inclusion and exclusion criteria during the period from December 2019 to September 2021. The study by Khatri et al. [10] observed an excellent result in 65.2% of patients (p=65.2). Considering an absolute error of 15% with a 95% confidence interval, the estimated sample size was 40 cases of volar Barton fracture. The Chi-square test was used for testing significance and a p-value <0.05 was considered statistically significant.

Immediate management

Following admission, history was elicited from the patient and attenders to reveal the mechanism of injury and the severity of trauma. All the patients were thoroughly examined. The general condition of the patient and associated systemic diseases and the injuries associated were noted. All the findings were recorded in the patient proforma. Careful inspection of the swelling, deformity, and evidence of ecchymosis were noted. Clinically tenderness, crepitus, abnormal mobility, distal sensation, and distal pulsation were assessed. Movements of the wrist were checked and found to be restricted due to pain. The involved forearm was immobilized with a below-elbow plaster of Paris (POP) slab and limb elevation was given. Pain and inflammation were managed using analgesics and anti-edema measures.

Preoperative planning

Routine blood investigations like complete blood count, renal function tests, bleeding and clotting time, blood grouping, and serology were sent. Blood pressure and ECG were recorded in all the patients. Preparation of parts was done for all the patients one day before surgery. Fitness for surgery was obtained for all the patients. Consent for surgery was taken from all the patients. Tetanus toxoid injection and prophylactic antibiotic were given pre-operatively.

Surgical procedure

Anesthesia

All the patients were operated on under general anesthesia or regional block.

Position and Torniquet

The patient was placed supine on the operating table. The affected arm was elevated for two to three minutes and exsanguinated using an Esmarch tourniquet. Then the mid-arm pneumatic tourniquet was applied. The limb was placed on the sidearm board by abducting the shoulder. The position of the limb should be in a way that allows complete imaging in the sagittal and coronal plane of the distal radius. Forearm and hand were thoroughly scrubbed, painted with betadine solution and spirit, and then draped.

Instruments and Implants Used

· Variable angle locking compression plates of varying lengths.

· 2.7mm LCP drill bit and sleeve system. 

· Hand drill/power drill.

· Tap for 3.5mm cortical screws and 3.5mm depth gauge.

· Hexagonal screwdriver for 2.7mm and 3.5mm locking screws and 3.5mm cortical screws.

· General instruments like retractors, periosteal elevators, reduction clamps, bone levers, etc

· Pneumatic tourniquet.

**Figure 1:**
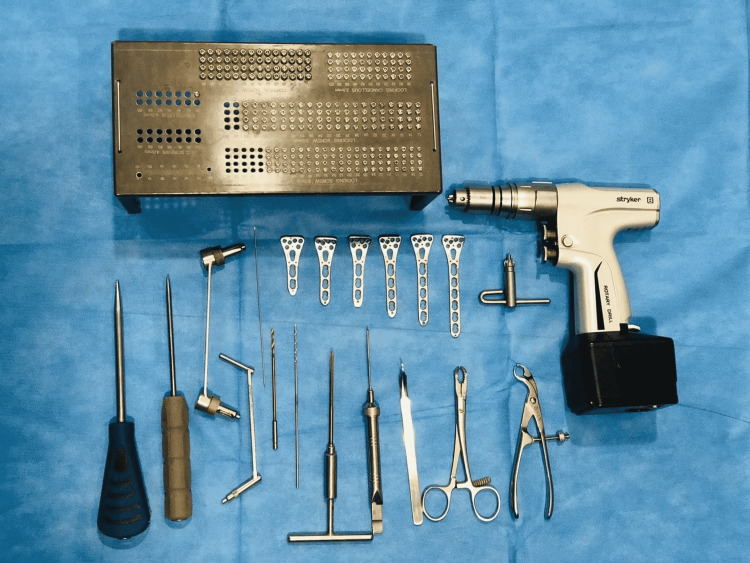
Instruments used

The instruments used are shown in Figure 1.

Operative procedure

Modified Henry’s Approach

The plane between the flexor carpi radialis and the radial artery is used in this approach. It is suitable for the fixation of most distal radius fractures. The modified Henry’s approach is ulnar to the radial artery. The incision is made along the radial border of the flexor carpi radialis tendon. The sheath is opened and the tendon is retracted towards the ulna. Adequate care was taken to prevent damage to the radial artery on the radial side and the palmar cutaneous nerve on the medial side. Flexor pollicis longus belly is swept towards the ulna by using the finger. Thus, the space is increased and the pronator quadratus muscle is exposed. Then the pronator quadratus muscle is incised using an L-shaped incision. The horizontal limb is placed over the watershed line. It lies a few millimeters proximal to the joint line. The pronator quadratus is incised on the radial border thus the distal radius is exposed. The muscle is stripped off from the distal radius together with the periosteum. The fracture line would be clearly visible now and reduced by manipulation and ligamentotaxis. Provisional K wires are used to hold the reduction. The appropriate plate with 3.5mm cortical and 4mm cancellous screws are placed. The screw size is checked under C arm guidance to prevent future complications. Pronator quadratus is sutured thus covering the distal end of the plate to prevent tendon irritation. Thus, the plate functions in two ways - to buttress the distal fragment and maintain the reduction of the metaphysis.

Postoperative protocol

Postoperatively, below-elbow functional slab was applied and the patients were advised to move the wrist after two weeks. Postoperative complications such as symptoms of median nerve compression, non-union, malunion, failure of fixation, wound infection, and Complex Regional Pain syndrome were assessed and documented. After discharge, patients were regularly followed up at one-month, three-month, and six-month intervals regularly. The range of motion was measured at every follow-up. The assessment of functional outcomes was made using the DASH Score and Gartland and Werley Demerit criteria. Radiological outcomes were assessed using Sarmiento modification of Lindstrom criteria.

## Results

A total of 40 subjects were included in the final analysis. The descriptive analysis of age is given in Table 1. Among the study population, 30 (75%) were male and the remaining 10 (25%) were female. Descriptive analysis of gender is given in Table 2.

**Table 1:** Descriptive analysis of age (in years) in study population (N=40) SD - Standard Deviation, CI - Confidence Interval

Parameter	Mean ± SD	Median	Minimum	Maximum	95% CI	
					Lower	Upper
Age (in years)	36.43 ± 10.59	35.50	20.00	58.00	33.04	39.81

**Table 2:** Descriptive analysis of gender in the study population (N=40)

Gender	Frequency	Percentages
Male	30	75.00%
Female	10	25.00%

Among modes of injury, nine patients (22.50%) had a history of fall from height and 31 patients (77.50%) had RTA. Twenty-four patients (60%) sustained an injury to the right side and 16 patients (40%) had a left-side injury. A descriptive analysis of the range of motion is given in Table 3.

**Table 3:** Descriptive analysis of the range of motion in the study population (N=40) SD - Standard Deviation

Parameter	Mean ± SD	Minimum	Maximum	
				Flexion	70.63° ± 3.6°	65.0°	78.0°	
Extension	74.3° ± 3.64°	68.0°	80.0°	
Radial Deviation	10.15° ± 1.55°	6.0°	12.0°	
Ulnar Deviation	30.58° ± 3.2°	25.0°	38.0°	
Supination	79.83° ± 2.67°	75.0°	85.0°	
Pronation	76.28° ± 1.99°	72.0°	80.0°	

The mean DASH score was 13.98 ± 5.76, ranging from 5 to 30, and the mean Gartland and Werley demerit criteria score was 2.73 ± 2.37, ranging from 0 to 10. Among the study population, 26 (65%) had excellent outcomes, 12 (30%) had good outcomes and two (5%) had fair outcomes according to Gartland and Werley's demerit criteria. The descriptive analysis is given in Table 4.

**Table 4:** Descriptive analysis of Gartland and Werley's outcome in the study population (N=40)

Gartland and Werley's outcome	Frequency	Percentages
Excellent	26	65.00%
Good	12	30.00%
Fair	2	5.00%

The mean loss of palmar tilt was 4.6° ± 3.75°, ranging from 0° to 12°, the mean radial shortening was 4mm ± 1.75mm ranging from 1mm to 7mm and the mean loss of radial inclination was 6.15° ± 2.83°, ranging from 0° to 12°. Thirty patients (75%) had an insignificant residual deformity, and 10 (25%) had a slight residual deformity. Among the study population, 22 patients (55%) had good radiological outcomes, 11 patients (27.50%) had excellent outcomes and seven patients (17.50%) had fair outcomes according to Sarmiento's modification of Lindstrom criteria. The descriptive analysis of the radiological outcomes of the study population is shown in Table 5.

**Table 5:** Descriptive analysis of radiological outcomes in the study population (N=40)

Radiological outcome	Frequency	Percentages
Good	22	55.00%
Excellent	11	27.50%
Fair	7	17.50%

Among the study population, two patients (5%) had a hypertrophic scar and superficial infection each. The superficial infection was treated with parenteral antibiotics according to the culture and sensitivity report, and one patient (2.50%) developed complex regional pain syndrome.

Keeping radiologically good outcomes as the baseline, the mean DASH score was 12.94 ± 5.32, 13.09 ± 5.41 in the excellent group, and 18.66 ± 6.05 in the fair group. Taking radiological outcome as the baseline, the mean difference in DASH score was statistically significant only in the fair group. Comparison of mean DASH scores across the study groups is given in Table 6.

**Table 6:** Comparison of mean DASH score across the study groups (N=40) SD - Standard Deviation, CI - Confidence Interval

Radiological outcome	DASH Score Mean ± SD	Mean difference	95% CI		p-value
			Lower	Upper	
Good	12.94 ± 5.32				
Excellent	13.09 ± 5.41	0.15	-3.94	4.25	0.939
Fair	18.66 ± 6.05	5.72	0.91	10.53	0.021

The mean Gartland and Werley demerit criteria score in the radiologically good outcome group was 2.32 ± 1.55, 1.82 ± 2.48 in the excellent group and 5.43 ± 2.7 in the fair group. Taking radiologically good outcomes as the baseline, the mean difference of Gartland and Werley demerit criteria score (0.50) in the excellent group was not statistically significant (p-value 0.514) and in the fair group (3.11) was statistically significant (p-value 0.001). Comparison of the mean Gartland and Werley demerit criteria scores across the study groups is given in Table 7.

**Table 7:** Comparison of mean Gartland and Werley demerit criteria score across the study groups (N=40) SD - Standard Deviation, CI - Confidence Interval

Radiological outcome	Gartland and Werley demerit criteria score Mean ± SD	Mean difference	95% CI		p-value
			Lower	Upper	
Good	2.32 ± 1.55				
Excellent	1.82 ± 2.48	0.50	-1.04	2.04	0.514
Fair	5.43 ± 2.7	3.11	1.30	4.92	0.001

## Discussion

Open reduction and internal fixation restore the wrist’s anatomy and help in faster rehabilitation with good clinical outcomes in cases of volar Barton fractures [11]. Volar plating is currently favored for comminuted distal end radius fracture patterns and osteoporotic bones [12]. The volar cortex of the distal end radius is often less comminuted than the dorsal cortex; therefore, anatomical reduction of the palmar cortex restores the radial shortening. Moreover, the palmar cortex is better contoured with respect to the dorsal cortex in terms of plate application. There had been a shift in focus from the use of non-locking volar plates to locking volar plates as the latter provides secure and reliable fixation of complex fractures due to angular stability [13]. Kanabar et al. [14] reported that early mobilization in fractures treated with volar fixed locking plates does not lead to a decrease in the radiological parameters achieved at the final follow-up. The present study aimed to assess functional outcomes in volar Barton fractures treated with variable locking plates by using the DASH score and Gartland and Werley demerit criteria and to assess the radiological outcomes using Sarmiento modification of Lindstrom criteria.

The present study involved 40 subjects with a mean age of 36.43 ± 10.59 years, ranging from 20 to 58 years. A majority of the study subjects were males (75%) and 25% were females. RTA was the major cause of injury in 77.50% and falls from height in 22.50%. Nearly 60% had right-side injuries and 40% had left-side injuries. A study by Kundu et al. [15] found that 30 patients were treated with volar locking plate system where 46.6% were male subjects and 53.33% were female and the average age was 42 years ranging from 18 to 64 years and had right-side injury in the majority. Another study by Kolla et al. [16] included 20 cases with volar Barton fractures with the majority of subjects in the 31 to 40 age group (45%) followed by 41 to 50 and 20 to 30 years at 25% each respectively. Males (80%) were affected more than females (20%) and the left side (65%) was more commonly injured than the right side (35%). RTAs (60%) were the most common cause of injury and 40% were due to Fall on An Outstretched Hand (FOOSH). 

**Figure 2:**
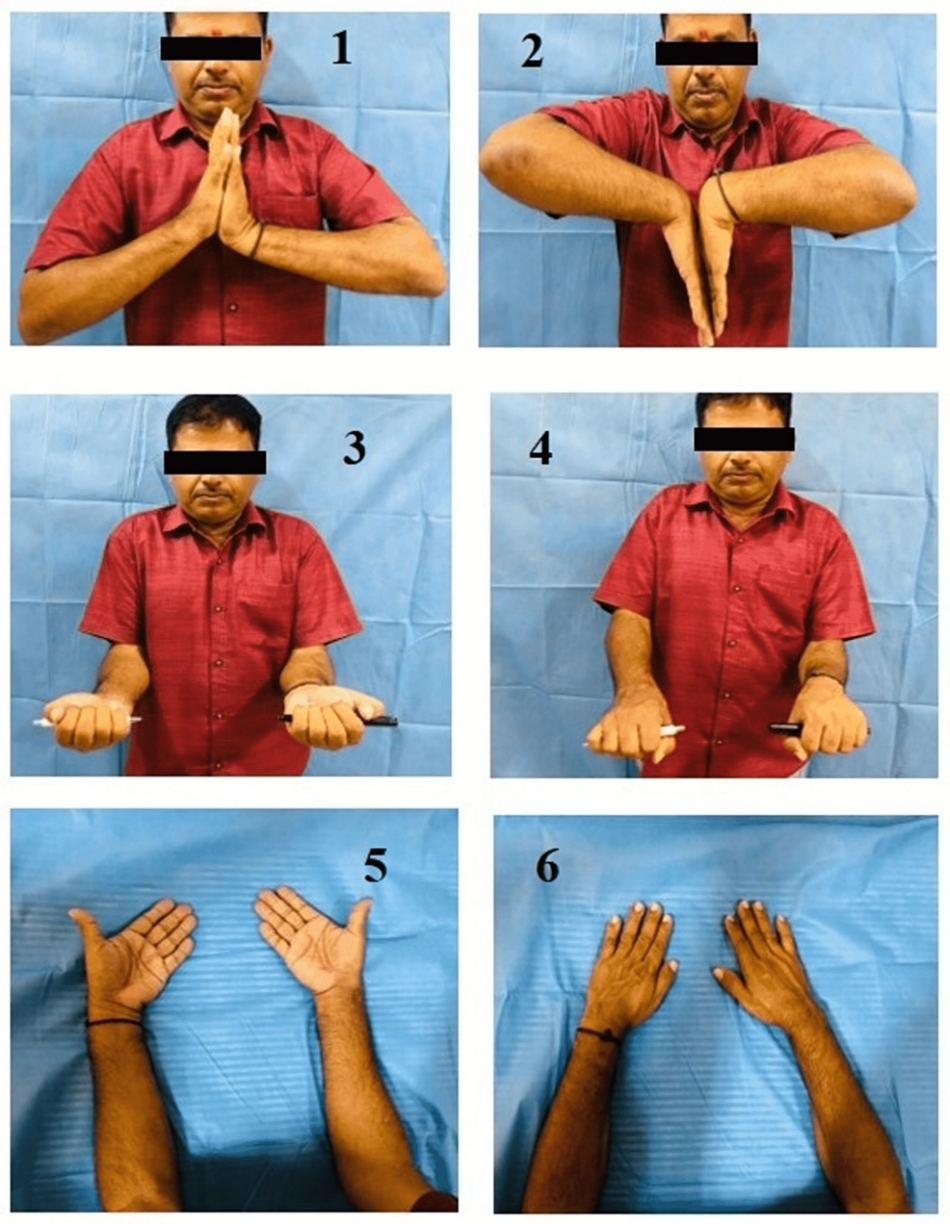
Range of motion measurement 1 - Dorsiflexion, 2 - Palmar flexion, 3 - Supination, 4 - Pronation, 5 - Ulnar deviation, 6 - Radial deviation

The present study observed that the mean flexion was 70.63° ± 3.6°, the mean extension was 74.3° ± 3.64°, the mean radial deviation was 10.15° ± 1.55°, the mean ulnar deviation was 30.58° ± 3.2°, the mean supination was 79.83° ± 2.67° and the mean pronation was 76.28° ± 1.99°. A study by Khatri et al. [10] at final follow-up found the mean flexion to be 71.91° ± 8.08°, the extension 76.95° ± 5.70°, pronation 77.65° ± 6.01°, and supination 81.86° ± 6.28°. The present study results were in comparison to the Khatri et al. study [10]. The range of motion measurement is shown in Figure 2.

**Figure 3:**
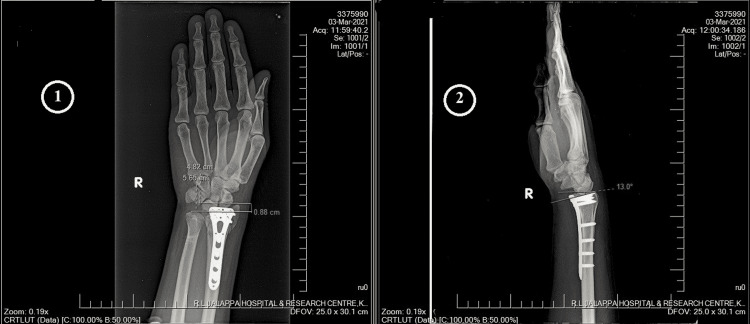
Radiological measurement 1 - Radial height, 2 - Volar tilt

The mean loss of palmar tilt was 4.6° ± 3.75°, ranging from 0° to 12°, the mean radial shortening was 4mm ± 1.75mm ranging from 1mm to 7mm and the mean loss of radial inclination was 6.15mm ± 2.83mm, ranging from 0mm to 12mm. A retrospective study by Khatri et al. [10] found the average radial inclination loss to be 0.68mm, radial length 0.1mm, the volar angle 0.26°, and ulnar variance 0.16mm, and found that the change in indices was statistically insignificant. Further, this study observed clinical parameters (flexion, extension, supination, and pronation) measured at eight weeks and at final follow-up revealed significant improvement. However, in our study, although we followed the patients at three and six months, we did not compare the mean scores at these intervals. In another study by Kundu et al. [15], at the final follow-up, the average volar tilt was 6.7° (range, 2° of dorsal tilt to 15° of volar tilt), radial inclination averaged 20.2° (range, 12-28°) and radial shortening averaged 0.7mm (range, 0-2mm). They found loss of volar tilt and trauma surgery interval correlating inversely to the functional outcome (p value<0.05). The radiological measurement is shown in Figure 3.

In the present study, the mean DASH score was 13.98 ± 5.76, and the mean Gartland and Werley demerit criteria score was 2.73 ± 2.37. Jagodzinski et al. [17] reported a mean DASH score of 18.2 in patients treated with variable angle locking compression plate (VA LCP). Based on Gartland and Werley's outcome, the majority (65%) of the study population had excellent outcomes, 30% had good outcomes and only 5% had fair outcomes. A similar result was found by Kolla et al. [16] with excellent Gartland and Werley outcomes in 55%, good in 35%, fair in 5%, and poor in 5% of their study population. Another study by Kundu A et al [15] with similar results to the present study found excellent outcomes in 70%, good in 16%, and fair in 14%.

Based on the anatomical and radiological evaluation using Sarmiento modification of Lindstrom criteria, we found the residual deformity, 30 (75%) had insignificant and 10 (25%) had slight. The study by Kundu et al. [15] found residual deformity to be excellent in 80%, good in 40%, and poor in 7%.

Among the study population, 22 (55%) had good radiological outcomes, 11 (27.50%) had excellent outcomes and seven (17.50%) had fair outcomes. The present study showed hypertrophic scar and superficial infection in 5% each and 2.50% had complex regional pain syndrome. Kolla et al. [16] found arthritis of the wrist joint in 15%, malunion fracture in 10%, and extensor pollicis longus tendon irritation in 5% due to long screw placement through the outer cortex irritating the tendon and one patient showed complex regional pain syndrome. The overall complication reported in Kundu et al. [15] was 18%. There were two cases of superficial wound infection that settled well with oral antibiotics. No patients had complex regional pain syndrome or non-union and none had extensor tendon irritation or ruptures. Residual pain, stiffness, and deformity were found in about 10% of the subjects.

In the present study, the mean DASH score within good radiological outcome was 12.94 ± 5.32, excellent was 13.09 ± 5.41 and fair was 18.66 ± 6.05. Taking good radiological outcome as a baseline, the mean difference of DASH score (0.15) in the excellent group was statistically not significant (p-value 0.939) and in the fair group (5.72) it was statistically significant (p-value 0.021). The mean Gartland and Werley demerit criteria scores within radiological good outcome were 2.32 ± 1.55, it was 1.82 ± 2.48 excellent and it was 5.43 ± 2.7 in fair. Taking radiologically good outcome as a baseline, the mean difference of Gartland and Werley demerit criteria score (0.50) in excellent was statistically not significant (p-value 0.514) and in fair (3.11) group, it was statistically significant (p-value 0.001). A retrospective study by Khatri et al. [10] observed Gartland and Werley’s demerit scoring system showed that 65.22% of subjects had excellent results while 34.78% had good results. A similar study by Figl et al. [18] reported excellent results in 37.5% of subjects, good results in 67%, and fair results in 1%. The results are, however, not truly comparable with those of the current study as a different scoring system was used in the evaluation of the results.

Limitations

The study was a single-center study with a limited sample size, limiting our results. A comparison with other modes of fixation was not done. The inclusion of a control group could have given a better comparison. Large multicentric studies with cases and control groups can further help in managing distal radius plating to prevent any complications and adverse outcomes.

## Conclusions

The present study was held to assess the functional and radiological outcomes of volar Barton fractures fixed with variable locking plates and the following points were concluded. The majority of them were male patients due to outdoor activities and RTAs. Most of the fractures occured in younger individuals due to high-velocity injuries. As per many studies, variable locking plates provide successful results for the treatment of distal radius fractures with intra-articular extension and are unstable in nature. Variable locking plates used for the management of volar Barton fractures provide efficient anatomic reduction, thus helping in early joint motion because of their rigid fixation. Modified Henry’s approach provides better accessibility with less surgical trauma while fixing volar Barton fractures with an adaption in a better way to the surrounding tissues. Most of the patients who were young adults returned to their daily activities comfortably.

We faced some complications during the study like a hypertrophic scar, superficial infection, and complex regional pain syndrome. But these complications were resolved and did not hinder the day-to-day activities of the patients in their final follow-up. The use of variable locking plates in volar Barton fractures provided good to excellent functional outcomes and excellent radiological outcomes in the majority of the patients. But the restoration of normal radiological parameters might not be necessary for the achievement of a better functional outcome.
